# Successful treatment of Cushing’s disease in a pediatric patient with Gamma Knife radiosurgery: a case report

**DOI:** 10.1097/MS9.0000000000003503

**Published:** 2025-06-16

**Authors:** Urooj Lal Rehman, Usama Ahmed Ansari, Khurram Khalid, Maliha Khalid, Marium Fatima, Muhammad Saad Khan, Erum Siddiqui, Aminath Waafira

**Affiliations:** a Department of Endocrinology (Medical Ward-6), Jinnah Post Graduate Medical Centre, Karachi, Pakistan; b FCPS, Jinnah Post Graduate Medical Centre, Karachi, Pakistan; c Department of Medicine, Jinnah Sindh Medical University, Karachi, Pakistan; d School of Medicine, The Maldives National University, Malé, Maldives

**Keywords:** adrenal insufficiency, Cushing’s disease, Gamma Knife radiosurgery, pituitary adenoma, young-onset endocrinology

## Abstract

**Introduction:**

Cushing’s disease in young-onset cases is a rare and diagnostically challenging endocrinopathy, most commonly caused by an adrenocorticotropic hormone (ACTH)-secreting pituitary adenoma. Early recognition and management are crucial to preventing long-term complications associated with hypercortisolism. This report highlights the case of a pediatric patient with Cushing’s disease, emphasizing diagnostic challenges and treatment considerations.

**Case presentation:**

We present the case of a 14-year-old male from Kashmore with a 4-year history of progressive truncal obesity, growth failure, and episodic headaches. Clinical evaluation revealed hallmark cushingoid features, including moon facies, dorsocervical and supraclavicular fat accumulation, acanthosis nigricans, and hypertension. A hormonal assessment confirmed hypercortisolism, and magnetic resonance imaging (MRI) revealed a 0.9 cm pituitary microadenoma.

**Clinical discussion:**

Cushing’s disease in pediatric patients presents unique diagnostic and therapeutic challenges due to its rarity and insidious onset. First-line treatment for pituitary-dependent Cushing’s disease is transsphenoidal surgery; however, in cases where surgery is declined or contraindicated, alternative therapies such as medical management and radiosurgery become viable options. In this case, Gamma Knife radiosurgery (GKRS) led to significant clinical improvements, including weight loss and biochemical remission. However, the patient later developed adrenal insufficiency, necessitating immediate steroid therapy, a known risk associated with successful treatment of hypercortisolism.

**Conclusion:**

GKRS has proven to be an effective treatment for Cushing’s disease in younger individuals, both as a standalone therapy and as an adjunct to other interventions. This case underscores the importance of individualized treatment approaches and close post-treatment monitoring for potential complications such as adrenal insufficiency.

## Introduction

Cushing syndrome (CS) is an endocrine disorder that occurs due to prolonged exposure to high levels of glucocorticoids and can be acquired exogenously or endogenously^[^1,2^]^. The overall incidence of endogenous CS is approximately 0.7–2.4 cases per million people per year. Cushing disease (CD), which results from an ACTH-producing pituitary tumor, accounts for 75–80% of endogenous CS cases^[^3^]^. It is the most common cause, representing 60–70% of cases in individuals under 18 and over 70% in adults^[^4^]^. In pediatric patients, multiple mechanisms contribute to the development of Cushing’s disease, reflecting its multifactorial nature. Enhanced EGFR signaling due to USP8 mutations is associated with increased ACTH secretion and the growth of corticotroph adenomas^[^5,6^]^. Furthermore, other genetic syndromes, including Multiple Endocrine Neoplasia type 1 (MEN1) and Carney complex, may predispose individuals to pituitary tumors. Environmental influences, though not well defined, are believed to interact with genetic predispositions to facilitate early tumor formation and hormonal disruption^[^7^]^. Younger patients with Cushing’s disease may experience a tougher clinical course, with surgery carrying more risks and the disease more likely to recur^[^8^]^. The primary treatment for CD is transsphenoidal surgery (TSS), which achieves remission rates of 71–78.9% in experienced centers^[^9^]^. In some cases, complete resection of the causative adenoma is not possible^[^10^]^. Furthermore, surgery increases the chance of pituitary insufficiency, which may necessitate lifelong hormone replacement therapy, which adds further complexity to disease management^[^11^]^. When surgery is not possible or does not lead to remission, other treatment options must be explored^[^12^]^. Gamma Knife radiosurgery (GKRS) has emerged as an effective treatment for recurrent or residual pituitary adenomas after surgery^[^13,14^]^. However, recent research suggests that GKRS could also be a primary treatment option for carefully selected CD patients who cannot have surgery or whose tumors cannot be removed. This method is a less invasive alternative, particularly for patients with elevated surgical risks or those deemed unfit for surgery^[^15^]^.

**HIGHLIGHTS**

- Rare pediatric Cushing’s disease: A 14-year-old male from Kashmore presented with a 4-year history of progressive truncal obesity, growth failure, and episodic headaches.
- Hallmark clinical features: The patient exhibited moon facies, dorsocervical and supraclavicular fat accumulation, acanthosis nigricans, and hypertension.
- Diagnostic confirmation: Hormonal assessments confirmed hypercortisolism, and MRI detected a 0.9 cm pituitary microadenoma.
- Treatment and outcome: Initially managed with ketoconazole, the patient opted for Gamma Knife radiosurgery (GKRS) over transsphenoidal surgery, leading to significant clinical improvement and biochemical remission.
- Post-treatment monitoring: The patient developed adrenal insufficiency requiring steroid therapy, but a six-month follow-up MRI confirmed complete tumor resolution.

## Case presentation

A 14-year-old male patient from Kashmore, Pakistan, with no known comorbidities, presented with progressive weight gain, short stature, and episodic headaches over 4 years. Despite maintaining a regular diet and physical activity, he had noticeable fat accumulation mainly around his trunk. He did not report any muscle weakness in the upper legs or arms, thinning of the skin, reddish stretch marks on the abdomen, or excessive body hair. There was also no history of frequent infections, back pain, walking difficulties, or vision problems. His family history was unremarkable, with no relatives known to have similar symptoms or chronic illnesses.

### Examination findings

**Figure 1.**
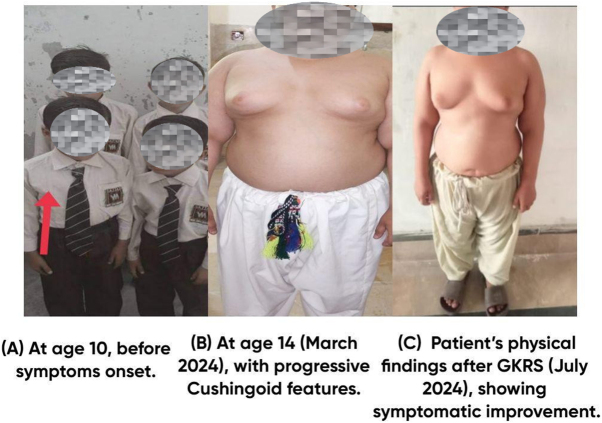
Physical findings.

**Figure 2.**
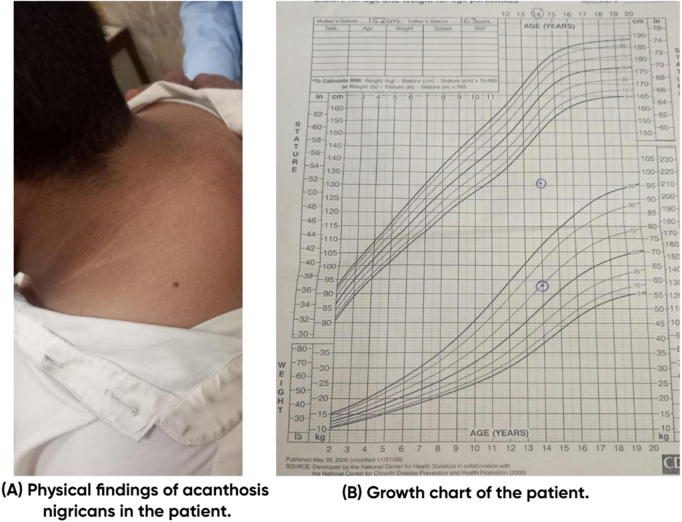
Acanthosis growth chart.

On physical examination, the patient exhibited short stature (131 cm, −3.2 SD for age) and obesity (weight: 58 kg, BMI: 34.3 kg/m^2^). His blood pressure was elevated (130/90 mmHg), with a heart rate of 100 bpm. Notable clinical features included a rounded plethoric face, dorsocervical and supraclavicular fat pads, bilateral adipomastia, and truncal obesity with a waist circumference of 94 cm. Palmar erythema and Acanthosis nigricans were observed, indicating underlying insulin resistance. Neurological assessment revealed normal intelligence using Wechsler Intelligence Scale for Children – Fifth Edition (WISC-V). On genital examination, the patient had sparse hair distribution with long, slightly pigmented straight hair at the base of the penis (Tanner stage 2). Scrotum was underdeveloped and Stretched Penile Length (SPL) was 1 inch. Testicles were soft and non-tender with bilateral volume of 2 ml. As shown in Figs. 1 and 2.

### Investigations

**Figure 3.**
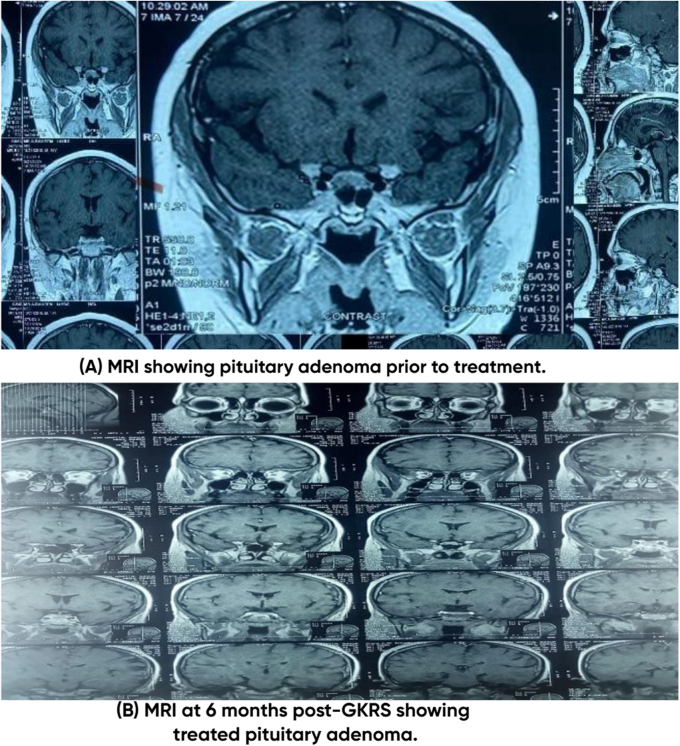
Pituitary adenoma pre and post GKRS.

Laboratory tests confirmed hypercortisolism and ACTH-dependent Cushing’s disease. The 1 mg overnight dexamethasone suppression test (ODST) showed cortisol at 4.10 µg/dL (>1.8 µg/dL), and 24-hour urinary free cortisol was markedly elevated at 591.09 µg/24 hr. Plasma ACTH was high (91 pg/mL, normal <46 pg/mL), and an 8 mg dexamethasone suppression test showed partial suppression (2.23 µg/dL). Hormonal evaluation indicated delayed pubertal development with low LH and FSH. A DEXA scan demonstrated osteoporosis in the lumbar spine (T-score: −4.1) and osteopenia in the left hip (T-score: −2.1), consistent with chronic cortisol excess. On MRI pituitary with contrast, a well-defined rounded lesion was seen at Pituitary fossa showing intermediate signal intensity on T1, T2, and FLAIR images and no obvious enhancement seen within it in post contrast images. It measured approximately 0.9 × 1.0 × 0.8 cm, suggestive of pituitary microadenoma. As illustrated in Fig. 3 and presented in Tables 1 and 2.

**Table 1:** Hematological, biochemical, and metabolic parameters

Parameter	Normal range	Before treatment	After treatment
Hemoglobin (Hb)	12–16 g/dL	12.6 g/dL	Not assessed
Sodium (Na^+^)	135–145 mmol/L	145 mmol/L	143 mmol/L
Potassium (K^+^)	3.5–5.1 mmol/L	4.2 mmol/L	3.1 mmol/L
Bicarbonate (HCO_3_^−^)	22–28 mmol/L	21.3 mmol/L	Not assessed
ALT	<45 IU/L	44 IU/L	Not assessed
AST	<35 IU/L	34 IU/L	Not assessed
ALP	44–147 IU/L	219 IU/L	Not assessed
Fasting blood sugar (FBS)	70–99 mg/dL	100 mg/dL	Not assessed
HbA1c	<5.7%	6.10%	Not assessed
Fasting insulin	2–25 mIU/L	34 mIU/L	Not assessed
HOMA-IR	<2.0	8.4	Not assessed
DEXA spine T-score	≥−1.0	−4.1	Not reassessed
DEXA hip T-score	≥−1.0	−2.1	Not reassessed

**Table 2:** Endocrine hormonal profile before and after treatment

Parameter	Normal range	Before treatment	After treatment
1 mg ODST	<1.8 µg/dL	4.10 µg/dL	Not assessed
24-hour urinary free cortisol	20.9–292.3 µg/24 hr	591.09 µg/24 hr	Not assessed
Plasma ACTH	<46 pg/mL	91 pg/mL	22.9 pg/mL
8 mg DST after dexamethasone	<5 µg/dL	2.23 µg/dL	Not assessed
Morning cortisol (8 am)	-	Not assessed	8.87 µg/dL
LH	1.2–7.8 mIU/mL	<0.3 mIU/mL	6.8 mIU/mL
FSH	0–10 mIU/mL	1.2 mIU/mL	5.28 mIU/mL
Testosterone	5–800 ng/dL	18 ng/dL	690 ng/dL
Serum prolactin	3–14.7 ng/mL	5.15 ng/mL	Not assessed
IGF-1	115.4–498.2 ng/mL	291.6 ng/mL	143.5 ng/mL

Diagnosis Cushing’s disease secondary to ACTH-secreting pituitary microadenoma.

### Management approach

The treatment strategy for this patient involved initial medical therapy with ketoconazole, an antifungal with steroidogenesis inhibition properties (200 mg) twice daily, with close monitoring of LFTs fortnightly, alongside metformin (500 mg) once daily for metabolic control and injection of zoledronic acid (4 mg) was administered over 30 mins for osteoporosis. Despite medical therapy, definitive intervention was required, leading to the decision for GKRS.

Although transsphenoidal surgery (TSS) is considered the first-line treatment for pituitary adenomas, the patient’s family declined surgical intervention after extensive counseling. Therefore, GKRS was selected as a non-invasive alternative. The expected outcome of GKRS was tumor growth control, reduction of cortisol hypersecretion, and eventual improvement in clinical symptoms. GKRS was considered an appropriate option due to its ability to precisely target the tumor while minimizing surrounding tissue damage and avoiding open surgery. This approach aligned with the family’s preference for a less invasive treatment modality. GKRS is increasingly recognized as a viable non-invasive option, especially when surgery is contraindicated or declined.

### Treatment and outcome

GKRS was performed on 5 April 2024, with a single fraction of 22 Gy fraction at a 50% isodose line, delivered via Leksell Gamma Unit Icon and was given Injection of Dexamethasone (8 mg) at the end of procedure. Post-procedure, oral Dexamethasone was administered and tapered over a month. Patient stopped tablet ketoconazole as advised by the radiosurgeon. Patient also failed to follow up in the endocrine clinic post procedure. The patient’s physical findings after GKRS, showing symptomatic improvement, are presented in Fig. 1.

### Post-treatment outcomes and challenges

At the three-month follow-up, the patient showed symptom improvement but developed persistent vomiting, hypotension (BP: 80/40 mmHg which corresponds to below the 5th percentile for his age and height), significant weight loss (45 kg, BMI: 26.2 kg/m^2^), waist Circumference (76 cm) and hypoglycemia (RBS: 74 mg/dL). Morning cortisol levels were low at 8.87 µg/dL, leading to a diagnosis of post-treatment adrenal insufficiency (AI). AI post-GKRS often signifies a successful treatment outcome but requires vigilant long-term monitoring to prevent adrenal crises. Patient was admitted and his baselines along with anterior pituitary hormones were sent. He was commenced on Injection hydrocortisone (HCT) (100 mg) intravenous (IV) three times a day and IV fluids. His vomiting subsided and he maintained a BP of 110/80 with a pulse of 74 beats/min. He was shifted to oral HCT (10 mg) at 7 am and (5 mg) at 5 pm daily and was doing well on current dose hence discharged from hospital. A six-month follow-up MRI showed no residual tumor, indicating a favorable response to treatment. Although the patient attended follow-ups at 3 and 6 months allowing assessment of symptoms and tumor status, he did not return for further endocrine clinic visits thereafter, limiting long-term evaluation of symptom recurrence, blood pressure percentiles, and adrenal recovery. This outcome is summarized in Fig. 3 and detailed in Tables 1 and 2.

## Discussion

Cushing’s disease (CD) in pediatric patients is an uncommon condition caused by adrenocorticotropic hormone (ACTH)-secreting pituitary adenomas, which results in excess cortisol production. Clinical features often include growth retardation, obesity, facial rounding, and delayed puberty. Diagnosis is established through biochemical testing, such as elevated urinary free cortisol and failed suppression on dexamethasone testing, along with pituitary imaging – typically MRI. Transsphenoidal surgery (TSS) is considered first-line therapy due to its potential for immediate remission, particularly in high-resource settings where neurosurgical expertise is readily available. In contrast, in low-resource settings where access to advanced neurosurgical care is limited – as in our current case – non-invasive approaches like stereotactic radiosurgery (SRS), including GKRS, may be favored as an initial treatment option.

Pituitary adenomas are among the most common intracranial primary tumors, representing as much as 10–20% of all brain tumors^[^16-18^]^. These mostly benign lesions can be classified as either nonfunctioning pituitary adenomas (NFPAs) or functioning pituitary adenomas (FPAs)^[^19,20^]^ based on clinically and biochemically evident endocrine secretory activity. With the exception of prolactinomas, most patients with symptomatic NFPAs and FPAs initially undergo resection. After resection, recurrence or progression of a known residual lesion can occur in as many as 10–50% of pituitary adenomas^[^21^]^. In FPA patients, such recurrence/progression can lead to a persistent hypersecretory state and related systemic morbidity. Historically, fractionated radiation therapy (RT) was used to treat patients with recurrent or progressive adenomas. However, over the past 2–3 decades, stereotactic radiosurgery (SRS), predominantly with the Gamma Knife, has been increasingly employed in the management of such patients^[^22-26^]^.

SRS is a non-invasive treatment method that delivers precise, high-dose radiation to targeted areas while sparing surrounding healthy tissue^[^27^]^. Two prominent SRS technologies are Gamma Knife and CyberKnife. Gamma Knife is designed for intracranial lesions and upper cervical spine treatment, while CyberKnife can treat both intracranial and extracranial sites^[^27^]^.

Hypopituitarism can arise as a result of iatrogenic injury to the normal neuroendocrine structures during treatments such as surgery, radiosurgery, and RT^[^28,29^]^. If undetected and uncorrected, hypopituitarism can have appreciable morbidity and even mortality. In one of the studies, we found that 24.2% of patients developed new-onset hypopituitarism following GKRS^[^30^]^, and this is comparable to rates published in several prior SRS reports^[^31,32^]^. This incidence is also lower than the historic risk of hypopituitarism following RT, which is believed to be as high as 50%^[^33^]^.

GKRS has emerged as an effective treatment for Cushing’s disease, particularly as an adjuvant therapy, with studies reporting remission rates ranging from 30% to 75%^[^27,34^]^. It is typically reserved for recurrent or persistent disease after failed surgery^[^35^]^. There is limited data on its effectiveness and long-term outcomes as the first-line therapy. In this case, GKRS was chosen as the primary treatment, which is unusual. The use of GKRS as the first-line treatment in an adolescent patient is even more uncommon since younger patients usually undergo surgery due to better recovery potential.

Tumor control rates are consistently high in GKRS, reaching 98–100%^[^27^]^. Factors influencing treatment success include tumor size, radiation dose, and discontinuation of cortisol-lowering medications during GKS^[^27,36^]^. Side effects are generally minimal, with new hypopituitarism occurring in 36% of patients in one study^[^27^]^. Visual improvements were noted in some cases^[^37^]^. In a study of eight patients, 6–18 years of age, submitted to stereotactic radiotherapy, all patients showed improvement of Cushing’s syndrome and seven of them went into full clinical remission. The urinary cortisol excretion returned to normal within the first month in five of the patients and a more gradual normalization was seen in two, taking 9–12 months to reach normal cortisol levels^[^38^]^.

In our case, the patient achieved remission but subsequently developed adrenal insufficiency, necessitating hydrocortisone replacement. This outcome aligns with existing literature, which acknowledges hypopituitarism as a potential delayed complication of GKRS. Notably, the incidence of such complications varies across studies like Esene *et al* observed no new cases^[^39^]^, while Gupta A. *et al*, in his multicenter international study^[^40^]^ also reports an 81% remission rate in CD patients, with a notable proportion developing hypopituitarism post-treatment, which aligns with this case’s outcome.

We planned the long-term follow-up for our patient, which is essential to monitor for adenoma recurrence, ensuring early detection of any regrowth. Additionally, regular surveillance of the hypothalamic-pituitary-adrenal (HPA) axis is necessary to assess recovery and adjust steroid therapy accordingly. Clinical and biochemical evaluations of anterior pituitary hormones, particularly growth hormone (GH), should be performed to detect any deficiencies. Once cortisol levels normalize, an insulin tolerance test (ITT) should be planned to evaluate GH secretion and determine the need for GH replacement therapy.

In addition to radiosurgery, pharmacological therapy with steroidogenesis inhibitors such as ketoconazole has been utilized in pediatric CD cases, particularly when surgery is delayed or not feasible. Ketoconazole inhibits adrenal cortisol synthesis by blocking cytochrome P450 enzymes (CYP11A1 and CYP11B1), and studies have reported biochemical control in a significant proportion of patients. Though not curative, ketoconazole can be a valuable bridge therapy or adjunct to definitive treatment options like GKRS or surgery.

This case is significant for its unique approach, which includes the use of GKRS as a first-line treatment for pediatric Cushing’s disease in a resource-limited setting. This contrasts with the existing literature, where GKRS is predominantly used post-surgically. Our case highlights the viability of GKRS as an alternative in centers where neurosurgical interventions are not feasible. It also reinforces the importance of endocrine follow-up to monitor for hypopituitarism, a frequent but manageable long-term complication. Thus, this case adds valuable clinical insight and broadens the scope of management strategies for pediatric CD in diverse healthcare environments. While the report mentions some post-treatment monitoring (e.g., MRI, HPA axis, GH evaluation), it lacks specific details such as recommended monitoring frequencies, clinical indicators, and protocols for managing complications like adrenal insufficiency. This limits its practical utility for clinicians treating pediatric Cushing’s disease.

## Conclusion

The management of Cushing’s disease in pediatric patients requires careful consideration of both treatment efficacy and long-term endocrine outcomes. In this case, GKRS provided disease control but resulted in adrenal insufficiency, underscoring the need for vigilant post-treatment monitoring. While GKRS may be a viable alternative for patients who decline surgery, conclusions about its broader effectiveness in younger populations cannot be drawn from a single case. Larger studies are needed to evaluate its long-term safety and role as a primary treatment option in pediatric Cushing’s disease.

## Data Availability

Not applicable.
